# Contrast‐enhanced ultrasound (CEUS) of benign and malignant renal tumors: Distinguishing CEUS features differ with tumor size

**DOI:** 10.1002/cam4.5101

**Published:** 2022-09-04

**Authors:** Jianing Zhu, Nan Li, Ping Zhao, Yanjie Wang, Qing Song, Luda Song, Qiuyang Li, Yukun Luo

**Affiliations:** ^1^ Department of Ultrasound, the First Medical Centre Chinese PLA General Hospital Beijing China; ^2^ Medical School of Chinese PLA Beijing China; ^3^ Department of Ultrasound, the Seventh Medical Centre Chinese PLA General Hospital Beijing China

**Keywords:** contrast‐enhanced ultrasound, imaging examinations, solid renal tumors, tumor size

## Abstract

**Background:**

Contrast‐enhanced ultrasound (CEUS) is now a guideline‐recommended strategy for diagnosing renal lesions. Tumor size is related to the risk of the treatment and prognosis in renal tumors. Thus, we aim to analyze the CEUS features of solid renal tumors in relation to tumor size.

**Methods:**

The CEUS appearance of 156 pathologically diagnosed solid renal tumors were retrospectively analyzed. Three groups were stratified according to the tumor size (≤2 cm [group I], 2.1–4 cm [group II] and 4.1–7 cm [group III]). For each group, the features of wash‐in type, enhancement degree, enhancement homogeneity, and the presence of a pseudocapsule sign were compared between benign and malignant tumors.

**Results:**

All 156 included lesions were detected by CEUS. The proportion of benign tumors in three size groups was 37.1%, 19.4%, and 20.4%, respectively. The proportion of malignant tumors was highest (80.6%) in group II, followed by group III (79.6%) and group I (62.9%). In group I, malignant and benign tumors differed significantly in the presence of a pseudocapsule sign (*p* = 0.015) and homogeneity (*p* = 0.007). In group II, the degree of enhancement differed (*p* = 0.02) between tumor types. In group III, the two tumor types differed in both the wash‐in pattern (*p* = 0.015) and enhancement degree (*p* = 0.024). The weighted and Cohen's kappa values for the concordance between inter‐observer agreement ranged from 0.31 (95% CI: 0.36–0.57) to 0.90 (95% CI: 0.77–1.00).

**Conclusions:**

CEUS features of malignant and benign renal tumors change along with the tumor size. The use of CEUS features in the diagnosis of benign and malignant tumors requires consideration of tumor size.

## Graphical abstract

This study shows that CEUS features of malignant and benign renal tumors change along with the tumor size. The use of CEUS features in the diagnosis of benign and malignant tumors requires consideration of tumor size.

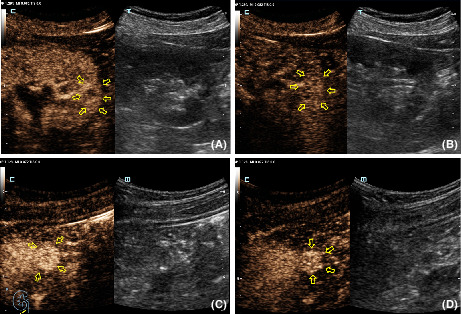

## 1 INTRODUCTION

Renal cell carcinoma (RCC) is the most lethal malignancy of the genitourinary system which stems from the tubular epithelium of the renal parenchyma,^1^ accounting for 80%–90% of renal malignancies.^2^ Solid localized renal tumors larger than 4 cm were defined as a large renal mass (LRM).^3^ According to the eighth edition (2017) of the TNM staging system, all histologic variants of RCC can be staged. In this system, tumors that are >4 cm but ≤7 cm along their longest axis and located to the kidney typically correspond to stage T1b RCC, while tumors >7 cm and limited to the kidney typically correspond to stage T2 RCC.^4^ Tumors smaller than 4 cm are defined as Small renal masses (SRM).^5^ These masses have TNM staging of T1a and can be further subdivided into two stages: T1a1 (≤2 cm) and T1a2 (2–4 cm), based on significantly different survival rates associated with different treatment (e.g., observation, radiofrequency thermal ablation, partial or total nephrectomy) for the two sizes.^6^ The incidence of RCC has increased by 3.7% per year.^7^, ^8^ Due to the improvement of imaging technology, the incidence of localized early T1 tumors increased the most.^9^ It is now widely accepted that most stage T1 RCCs have a favorable outcome.^7^ Tang et al.^10^ reported that RCCs in substage T1a1 had significantly better prognosis than T1a2. In a research of 2770 pathologically renal tumors, the proportion of benign masses was inversely proportional to their size; 46.3% of tumors smaller than 1 cm were benign, while only 6.3% of tumors larger than 7 cm were benign.^11^ Because of these reasons, most previous studies on the characteristics of renal tumors have focused on the differentiation of benign and malignant tumors.

Contrast‐enhanced computer tomography (CECT) remains the current diagnostic standard,^1^ but major limitations exist, such as radiation exposure, contrast agent allergy and contrast agent nephrotoxicity.^12^ Contrast‐enhanced magnetic resonance imaging (CEMR) was recommended for diagnosing renal tumors according to the European Association of Urology (EAU) guidelines.^1^ However, the limitations include expensive costs, claustrophobic, the longer examination time and gadolinium contrast agent allergies may prevent use of CEMR examination.^13^, ^14^ CT and MRI can not accurately distinguish between benign and malignant renal masses, with the exception of lipid‐rich angiomyolipoma (AML).^15^ Preliminary studies have suggested that for diagnosing renal masses, contrast‐enhanced ultrasound (CEUS) may be at least equal to CECT and CEMR.^14^ In a study involving 36 unclear solid renal tumors, Rübenthaler et al.^16^ found that CEUS had better specificity, ROC area, positive predictive value (PPV), negative predictive value (NPV) and diagnostic accuracy than CEMR, and equal sensitivity to CEMR with regard to different between benign and malignant renal tumors. However, no research to date has systematically described the correlation between tumor size and CEUS features. Thus, the purpose of this study is to analyze the possible relationship between the two.

## 2 PATIENTS AND METHODS

### 2.1 Patients

**FIGURE 1:**
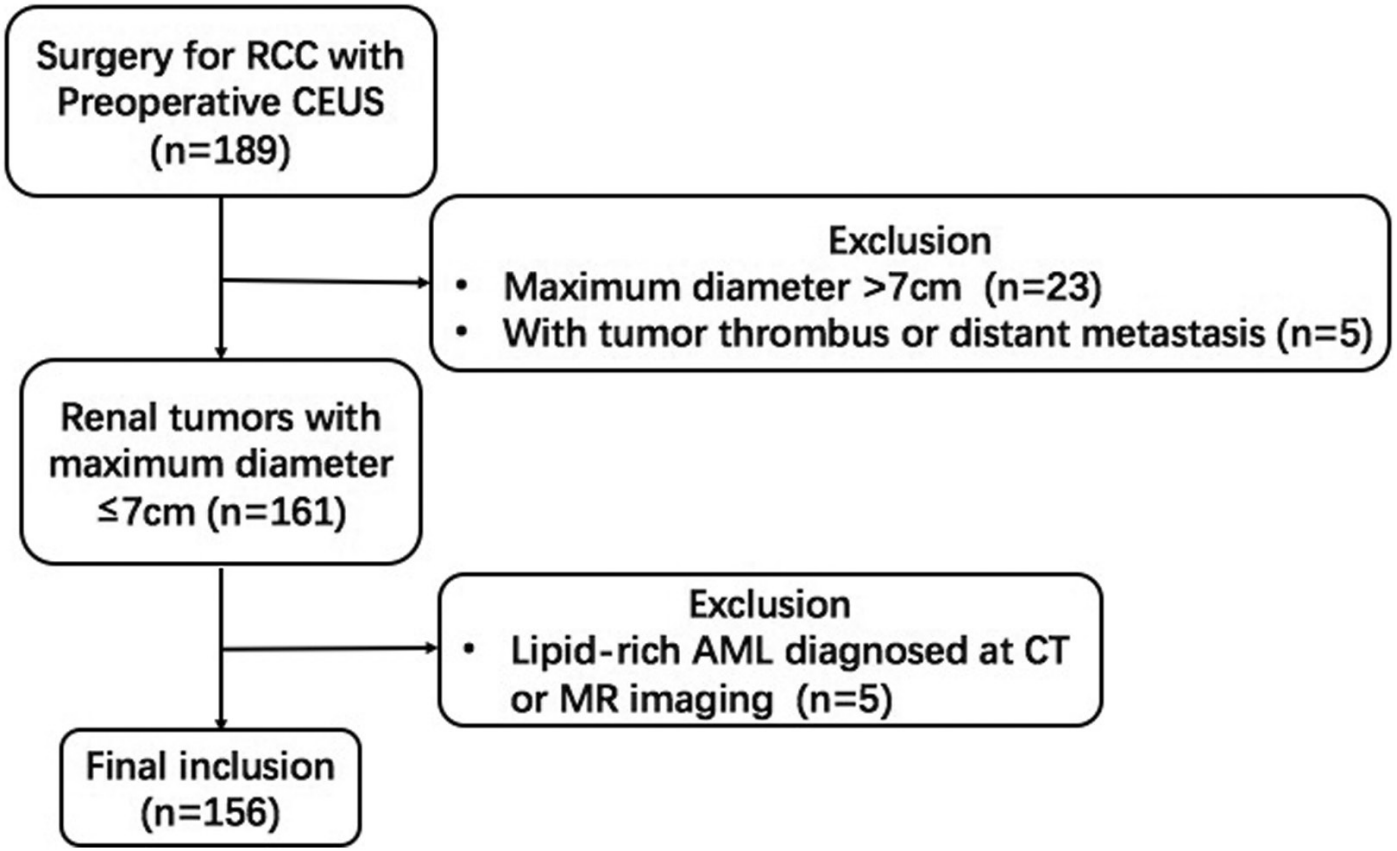
Patient flow diagram.

We retrospectively reviewed 156 renal masses selected from 155 patients who underwent preoperative ultrasound examination at a medical center in Beijing, China between January 2018 and April 2022; patients included 103 males and 52 females. The inclusion criteria were as follows: (a) over 18 years of age; (b) a solid mass diagnosed with CT, MR imaging, or CEUS; (c) a biopsy‐ or surgery‐proved diagnosis; and (d) the maximum diameter of the tumor was ≤7 cm as diagnosed by conventional ultrasound. Exclusion criteria include: (a) pregnancy; (b) lipid‐rich AML confirmed with CT or MRI; and (c) the presence of tumor thrombus or distant metastasis (Figure 1). Most patients involved had no symptoms, with tumors found during a routine physical examination; the exceptions were 13 patients with waist pain, 16 patients with painless gross hematuria, and three patients with frequency and urgency of urination. The study was supported by the Ethics Committee and Institutional Review Board of Chinese PLA General hospital (S2021‐302‐01). As this was a retrospective study that did not involve private patient information, the data did not require informed consent of patients. Prior to the contrast‐enhanced ultrasound examination, all patients gave their written informed consent.

### 2.2 Ultrasonographic examination

All cases in this study had an ultrasound (US) performed using an SC6‐1 probe (Mindray Resona 7). The CEUS characterizations of renal tumors were performed in a stepwise approach. First, conventional Ultrasound examination (two‐dimensional and color Doppler US) was used to localize the lesion. Then, the suspension SonoVue (Bracco®) was quickly injected via a peripheral ante cubital vein with a bolus of 1.0–1.2 ml based on the patient's weight and tumor size. An extremely low mechanical index (MI = 0.05–0.08) was used. Following bolus injection, each examination lasted at least 3 min. Continuous dynamic observation of the masses under angiography was processed for 3 min and images were preserved.

### 2.3 Review process

Tumors were stratified into three groups based on size, as measured from ultrasonography: tumors ≤2 cm in diameter (group I), tumors 2.1–4 cm (group II), and tumors 4.1–7 cm (group III). In our retrospective review of the cases, we found that all pathologically confirmed benign tumors with a maximum diameter greater than 7 cm could be identified as lipid‐rich AMLs with typical benign characteristics on CT or MRI, so we excluded all benign tumors >7 cm from the study cohort. Therefore, Tumors larger than 7 cm were not included in this study as no benign control tumors were present in this subgroup.

Video clips of qualitative imaging were reviewed separately by two reviewers that were blinded to the final pathologic diagnosis. Reviewer 1 (Q.Y.L.) had 15 years of experience with abdominal CEUS. When evaluation results differed, the two reviewers re‐visited results and reached a consensus. Wash‐in types (i.e., fast, simultaneous, or slow), enhancement level (i.e., hypo‐, iso‐, or hyper‐enhancement), enhancement homogeneity, and the presence of a pseudocapsule were evaluated. Because this examination occurs in real time, the presence of wash‐in and the degree of enhancement relative to the surrounding parenchyma could be compared. Heterogeneous enhancement is defined as enhancement that varies in different areas of the lesion.^7^ Pseudocapsule sign refers to a rim surrounding the tumor with the enhancement higher than that of the lesion and adjacent cortex.

### 2.4 Statistical analysis

All statistical data were analyzed using IBM SPSS 26.0 (SPSS Inc). Measured data are reported as mean ± standard deviation (SD) and categorical data are reported as frequency or percentage (%). Clinical, pathologic, and imaging variables were compared using a Mann–Whitney U test, a Student's *t*‐test, a Chi‐square test, or a Fisher's exact test. A two‐tailed *p* < 0.05 was considered statistically significant.

Inter‐rater reliability was assessed by MedCalc version 12.5 (MedCalc Software) using weighted *κ* or Cohen *κ* statistics. An *κ* value of greater than 0.75 was considered as excellent; 0.40 to 0.74 as good; and <0.39 as poor.

## 3 RESULTS

The demographics of patients with benign and malignant lesions are summarized in Table 1. The study population consisted of 156 tumors in 155 patients. There was no statistical significance of the mean age between benign and malignant tumors in three groups (*p* = 0.637, 0.054, 0.106 for groups I, II and III, respectively) or the mean maximum diameter of tumors (*p* = 0.628, 0.095, 0.095) among the three groups. There were more men than women in the study cohort (*p* = 0.005), but sex ratio of malignant and benign tumors did not differ for any of the three groups (*p* = 0.510, >0.999, 0.482 for groups I, II and III, respectively).

**TABLE 1:** Demographics of benign and malignant lesions

Characteristic	≤2 cm		2.1–4 cm		4.1–7 cm	
	Malignant (*n* = 22)	Benign (*n* = 13)	Malignant (*n* = 58)	Benign (*n* = 14)	Malignant (*n* = 39)	Benign (*n* = 10)
Age (y)	55 ± 10.78	50 ± 14.63	55 ± 11.72	47 ± 13.24	53 ± 12.49	47 ± 12.72
*p* value^a^	0.64		0.05		0.11	
Sex^b^						
Male	17 (77.3)	5 (38.5)	46 (79.3)	6 (42.9)	25 (64.1)	4 (40.0)
Female	5 (22.7)	8 (61.5)	11 (19.1)	8 (57.1)	13 (33.3)	6 (60.0)
*p* value^c^	0.51		1.00		0.48	
*D*_max_ (cm)^d^	1.55 (1.4–1.7)	1.50 (1.4–1.9)	3.15 (2.5–3.4)	2.65 (2.2–3.3)	5.30 (4.4–5.9)	5.20 (4.7–5.9)
*p* value^e^	0.63		0.09		0.09	

Abbreviation: *D*_max_, Mean maximum diameter of tumors.

^a^ Student's *t*‐test.

^b^ Data are numbers of patients. And numbers in parentheses are percentage (%).

^c^ Chi‐square test.

^d^ Numbers in parentheses are a range.

^e^ Mann–Whitney *U* test.

Among the 37 histologically confirmed benign renal lesions, the proportion of benign tumors in group I was the highest (13/35, 37.1%), followed by group II (14/72, 19.4%) and then group III (10/49, 20.4%). Among all benign tumors, lipid‐poor AML tumors were found in seven (of 13), nine (of 14), and four (of 10) cases in groups I to III, respectively. Groups I, II, and III had three, two and two cases, respectively of renal oncocytomas. Other benign lesion counts were three, three and four cases in groups I to III, respectively. One patient had two AML tumors evaluated; CEUS was performed on the same day with a 15‐min interval. The distribution of benign tumor histological subtypes did not differ between the three groups (*χ* ^2^ = 7.935, *p* = 0.635; Table 2).

**TABLE 2:** Pathological types and histological subtypes of renal tumors of different sizes

Histological types	Group (*n*)		
	I (*n* = 35)	II (*n* = 72)	III (*n* = 49)
Malignant			
Clear cell renal cell carcinoma	20 (57.1)	43 (59.7)	23 (46.9)
Chromophobe cell carcinoma	1 (2.8)	5 (6.9)	11 (22.4)
Papillary cell carcinoma	1 (2.8)	9 (12.5)	5 (10.2)
Mucinous tubular and spindle cell carcinoma	0 (0.0)	1 (1.3)	0 (0.0)
Benign			
Renal oncocytoma	3 (8.5)	2 (2.8)	2 (4.1)
Angiomyolipoma^a^	7 (20.0)	9 (12.5)	4 (8.2)
PEComa	1 (2.8)	2 (2.8)	2 (4.1)
Inflammatory pseudotumor	0 (0.0)	1 (1.3)	0 (0.0)
Anastomosing hemangioma	1 (2.8)	0 (0.0)	0 (0.0)
Juxtaglomerular cell tumor	1 (2.8)	0 (0.0)	2 (4.1)

*Note*: Data are numbers of patients. And numbers in parentheses are percentage (%).

Abbreviation: PEComa, perivascular epithelioid cell tumor.

^a^ Lipid‐poor angiomyolipoma.

For the 119 malignant renal lesions observed, group II had the highest proportion of malignant tumors (58/72, 80.6%), followed by group III (39/49,79.6%) and then group I (22/35, 62.9%). Clear cell renal cell carcinoma (ccRCC) accounted for 20/35, 43/72, and 23/49 malignant tumors, in groups I to III, respectively. Chromophobe cell carcinoma (chRCC) was most common in group III (11/49, 47.8%) and papillary cell carcinoma (pRCC) was most common in group II (9/72, 12.5%). The distribution of malignant tumor histological subtypes differed significantly among size groups (*χ* ^2^ = 12.670, *p* = 0.049; Table 2).

**FIGURE 2:**
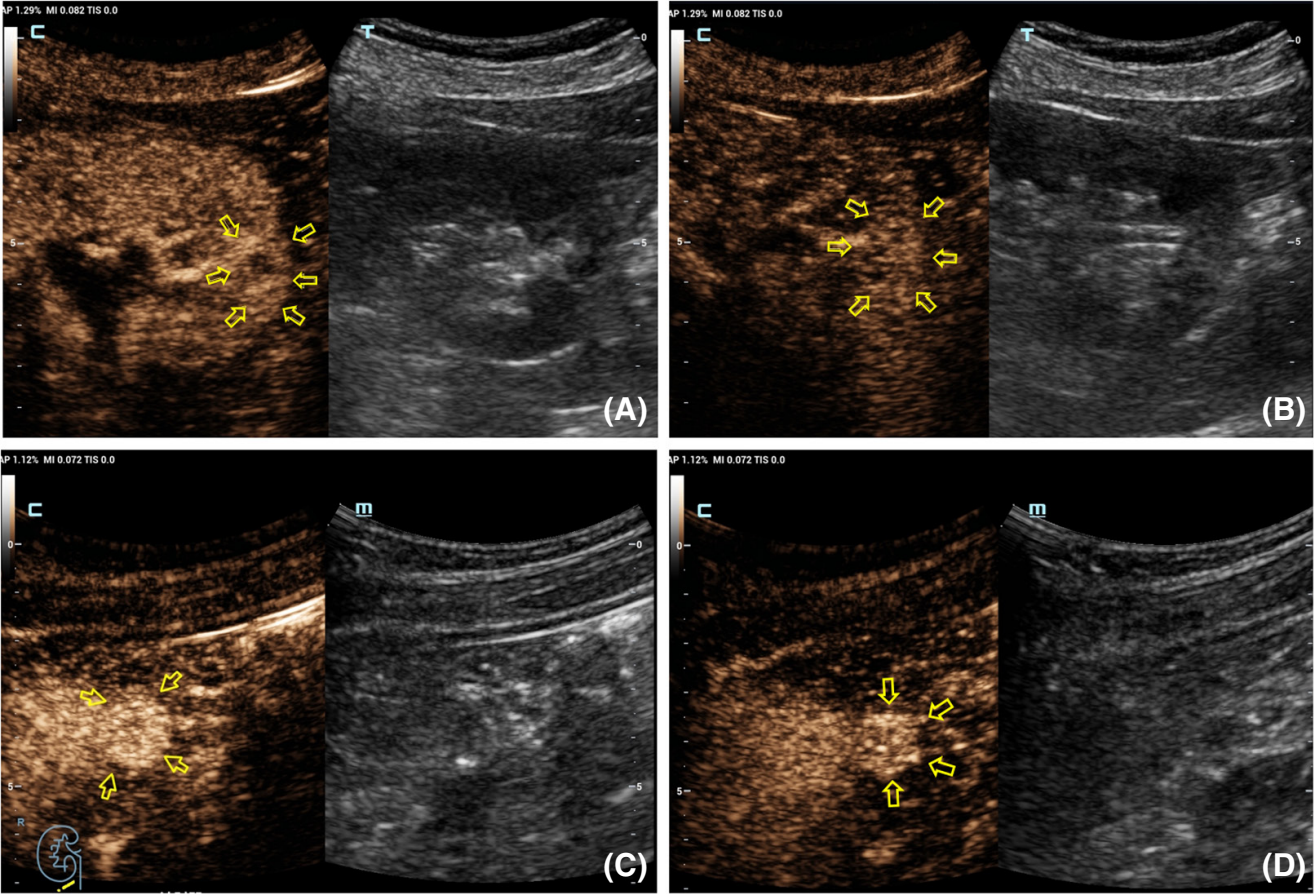
(A, B) A 76‐year‐old woman. CEUS shows a 1.5 cm size solid tumor with high‐ and heterogeneous enhancement in 25 s (A) and 1 min 45 s (B), respectively. Pathology revealed ccRCC, WHO/ISUP grade I. (C, D) A 29‐year‐old women. CEUS shows a 1.5 cm size solid tumor with high‐ and homogeneous enhancement in 27 s (C) and 1 min 50 s (D), respectively. Pathology revealed lipid‐poor AML. No pseudocapsule can be observed around either tumors.

**FIGURE 3:**
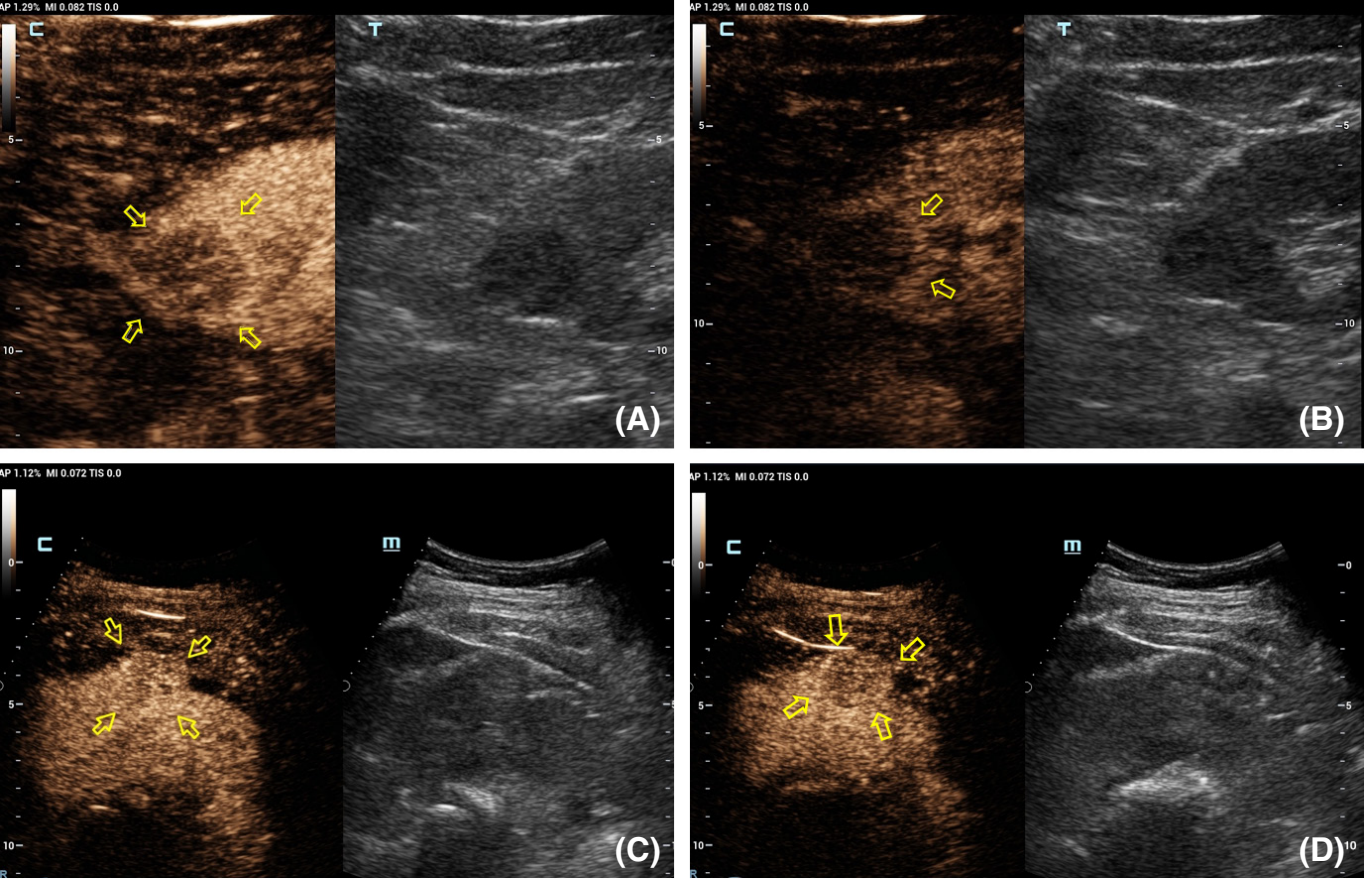
(A, B) A 47‐year‐old man. CEUS shows a 2.5 cm size solid tumor with low‐ and heterogeneous enhancementin 36 s (A) and 2 min 5 s (B), respectively. An irregular pseudocapsule was observed around tumor. Pathology revealed ccRCC, WHO/ISUP grade II. (C, D) A 40‐year‐old women. CEUS shows a 3.5 cm size solid tumor with iso‐ and homogeneous enhancement in 33 s (C) and 1 min 46 s (D), respectively. Pathology revealed PEComa

Table 2 shows the CEUS features observed for different tumor size groups. In group I, the presence of homogeneity and a pseudocapsule sign differed significantly between benign and malignant renal lesions (*p* = 0.015 and 0.024, respectively; Table 4). Inter‐reader variability was excellent for wash‐in type (*κ* = 0.64) and good for the other three CEUS features (*κ* = 0.41, 0.42 and 0.44 for enhancement level, homogeneity, and presence of a post‐contrast halo, respectively; Figure 2). In group II, the only different feature between benign and malignant tumors was the degree of enhancement (*p* = 0.02; Table 4). Hyper‐enhancement in the arterial phase (8–35 s) of the tumor was always associated with a malignant lesion, while iso‐enhancement was always observed with a benign lesion. Other CEUS findings, such as wash‐in, the presence of the pseudocapsule sign and homogeneity, did not differ statistically between benign and malignant tumors (*p* > 0.05; Table 4, Figure 3). Inter‐reader variability of wash‐in and enhancement degree ranged from 0.69 to 0.90, indicating excellent, while the presence of pseudocapsule was moderate and the homogeneity was low (*κ* = 0.41 and 0.31, respectively) (Table 3). In group III, malignant and benign tumors differed significantly for wash‐in pattern and enhancement level (*p* = 0.015 and 0.024, respectively). The two CEUS features had an excellent inter‐reader agreement (all *κ* > 0.71), as did homogeneity (Table 3).

**TABLE 3:** Comparison of different CEUS features in different tumor sizes of benign and malignant lesions for reviewer 1 and reviewer 2 and their interobserver agreement

CEUS feature	Group (*n*)					
	I		II		III	
	Malignant (*n* = 22)^a^	Benign (*n* = 13)^a^	Malignant (*n* = 58)^a^	Benign (*n* = 14)^a^	Malignant (*n* = 39)^a^	Benign (*n* = 10)^a^
Wash‐in						
Fast	20.19	9.8	50.48	9.7	34.33	6.5
Simultaneous	2.3	4.5	5.8	2.4	4.5	4.5
Slow	0.0	0.0	3.4	3.3	1.1	0.0
Weighted *κ* ^b^	0.64 (0.32–0.96)		0.90 (0.77–1.00)		0.80 (0.63–0.98)	
Enhancement						
Hyper‐enhancement	20.13	11.9	47.39	9.6	29.24	4.4
Iso‐enhancement	0.6	2.4	1.9	2.7	3.9	0.0
Hypo‐enhancement	2.3	0.0	10.10	3.1	7.6	6.6
Weighted *κ* ^b^	0.44 (0.14–0.73)		0.69 (0.50–0.89)		0.77 (0.63–0.91)	
Pseudocapsule						
Present	15.13	7.5	48.50	7.5	34.30	6.6
Absent	7.9	6.8	10.8	7.9	5.9	4.4
Cohen *κ* ^b^	0.41 (0.10–0.71)		0.41 (0.12–0.70)		0.37 (0.10–0.64)	
Homogeneity						
Present	6.8	7.5	13.15	7.10	9.11	2.4
Absent	16.14	6.8	45.43	7.6	30.28	8.6
Cohen *κ* ^b^	0.42 (0.11–0.74)		0.31 (0.36–0.59)		0.53 (0.30–0.77)	

^a^ Data are number of lesions for reviewer 1 and reviewer 2, respectively.

^b^ Data in parentheses are 95% CIs.

**TABLE 4:** CEUS features for benign and malignant lesions in different tumor sizes

CEUS feature	Group (*n*)					
	I		II		III	
	Malignant (*n* = 22)^a^	Benign (*n* = 13)^a^	Malignant (*n* = 58)^a^	Benign (*n* = 14)^a^	Malignant (*n* = 39)^a^	Benign (*n* = 10)^a^
Wash‐in						
Fast	19 (86.4)	8 (61.5)	50 (86.2)	9 (64.3)	34 (87.2)	6 (60.0)
Simultaneous	3 (13.6)	5 (38.5)	5 (8.6)	2 (14.3)	4 (10.3)	4 (40.0)
Slow	0 (0.0)	0 (0.0)	3 (5.2)	3 (21.4)	1 (2.5)	0 (0.0)
*p* value	0.51		0.35		0.02	
Enhancement						
Hyper‐enhancement	20 (90.9)	11 (84.6)	39 (67.2)	6 (42.9)	29 (74.4)	4 (40.0)
Iso‐enhancement	0 (0.0)	2 (15.4)	9 (15.5)	7 (50.0)	3 (7.7)	0 (0.0)
Hypo‐enhancement	2 (9.9)	0 (0.0)	10 (17.2)	1 (7.1)	7 (17.9)	6 (60.0)
*p* value	0.42		0.02		0.02	
Pseudocapsule						
Present	15 (68.2)	3 (23.1)	46 (79.3)	9 (64.3)	28 (71.8)	6 (60.0)
Absent	7 (31.8)	10 (76.9)	12 (20.7)	5 (35.7)	11 (28.2)	4 (40.0)
*p* value	0.02		0.30^b^		0.47	
Homogeneity						
Present	4 (18.2)	8 (61.5)	15 (25.9)	10 (71.4)	11 (28.2)	4 (40.0)
Absent	18 (81.8)	5 (38.5)	43 (74.1)	6 (42.9)	28 (71.8)	6 (60.0)
*p* value	0.02		0.05		0.47	

^a^ Data in parentheses are percentage (%).

^b^ Fisher's exact test; others: Chi‐square test.

The pseudocapsule sign was found in 59.1%, 79.3%, and 71.8% of malignant tumors and in 30.7%, 64.3%, and 60.0% of benign tumors in groups I to III, respectively. When benign and malignant tumors are combined, the presence of the pseudocapsule sign was higher in group II than in group I or group III tumors, although differences are not statistically significant (*p* = 0.186 and 0.179, respectively). Homogeneity among size groups was not statistically significantly different in either malignant or benign tumors (*p* = 0.679 and 0.534, respectively).

## 4 DISCUSSION

Solid renal masses without macroscopic fat are difficult to differentiate by type with imaging, especially when the tumor size is small. Yet preoperative imaging diagnosis directly affects further clinical decisions. Within the literature guidelines^17^ (AUA, EAU), elderly patients with underlying diseases who incidentally diagnosed with SRM have a low RCC‐specific mortality, and thus active surveillance (AS) can be an attractive treatment strategy.^18^ Renal tumors up to 3 cm in diameter, including asymptomatic tumors, always with a higher nuclear grade and a possibility of extraperitoneal invasion of the renal capsule,^19^ even though these tumors are still considered at stage T1a, they are highly possible to progress.^10^ In our series, benign tumors accounted for 37.1%, 19.5%, and 20.4% in size groups I‐III, respectively. This suggests that the percentage of benign tumors is lower when the tumor diameter is larger than 2 cm, in agreement with Xiong et al.^20^

The distribution of histological subtypes varies with tumor size in malignant tumors. The proportion of ccRCC and pRCC was higher in 2.1–4 cm tumors and lower in >4 cm tumors, while the proportion of chRCC increased with size. This trend is partially inconsistent with the results of foreign studies. Schachter et al.^21^ found fewer ccRCC and more pRCC in renal carcinoma tumors ≤4 cm, compared to tumors >4 cm. Rothman et al.^22^ reviewed the relationship between the incidence of localized renal carcinoma subtypes and tumor diameter in 19,932 cases and found a convex curve for ccRCC and a concave curve for pRCC. Differences between our study and these previous studies may be related to the target population, which needs to be further verified by larger samples.

We evaluated several CEUS features to assess the value of CEUS for distinguishing benign and malignant solid SRMs of different diameters. The main difficulty when imaging renal masses are differentiating lipid‐poor AML from ccRCC and oncocytoma from ChRCC. Among all malignant tumors in this study, most ccRCC in were hyper‐enhanced and had earlier wash‐in compared to renal parenchyma. In the 86 ccRCCs in our study, 83 ccRCCs were identified by CEUS; three tumors were misdiagnosed as non‐ccRCC, two in group I and one in group II. In the 33 non‐ccRCCs, 30 cases were identified by CEUS; one case of pRCC was misdiagnosed as AML in each of groups I and II, and a mucinous tubular and spindle cell carcinoma (MTSCC) in group II was misdiagnosed as chRCC. In the latter case of MTSCC, the CEUS of the tumor was characterized by slow wash‐in, hypo‐enhancement, with a pseudocapsule sign. The hypovascular feature of MTSCC may be related to the close arrangement of tubular cells and spindle cells with myxoid stroma in the tumor.^23^ As MTSCC tumor cells are well differentiated and have a low degree of malignancy, the tumor is expansionary and often compresses adjacent renal parenchyma to form a fibrotic pseudocapsule.^24^

Classic (lipid‐rich) AMLs are readily identified using conventional US due to their macroscopic fat, a feature rarely seen in other solid renal tumors. However, lipid‐poor AMLs are generally considered difficult to distinguish from RCC or oncocytomas at imaging due to their similar imaging findings.^25^, ^26^ In the 20 AMLs of our study, seven cases (100%) in group I, five cases (71%) in group II and one case (25%) in group III were misdiagnosed as RCCs. Previous work has suggested that slow centripetal and heterogeneity enhancement are characteristic CEUS findings for lipid‐poor AMLs.^27^ In our study, only one case in each of groups II and III presented with those CEUS features and, in group I, this feature was not found in any cases. This result may indicate that this feature is related to tumor size.

Oncocytomas are confirmed as benign tumors with low metastatic potential but with a similar growth rate to RCC.^28^ Not only is imaging diagnosis of oncocytomas difficult,^29^, ^30^ but pathological differential diagnosis from chRCC can be difficult.^31^ Thus, oncocytomas were often misdiagnosed and are often removed surgically. In the present study, two (66.7%) oncocytoma in group I and one (50%) in group II were misdiagnosed as RCC. The remaining oncocytoma (one in each of groups I and II) were identified by CEUS, based on the presence of centripetal enhancement, a spoke‐wheel pattern of enhancement present in a minority of oncocytoma cases.^32^

In our study, presence of the pseudocapsule sign occurred more frequently in 2.1–4 cm tumors than in tumors ≤2 cm or 4.1–7 cm, in both malignant and benign tumors. Jun et al.^33^ reported that a pseudocapsule was more frequently shown in tumors sized 2.1–5 cm than in tumors <2 or >5 cm, consistent with our finding. This may occur, because in tumors <2 cm, the pseudocapsule is so thin that can not be found or unformed due to the short growth period. While in tumors sized 2.1–4 cm, the tumor has had sufficient growth time to stimulate reactive hyperplasia of the renal interstitial fibrous tissue and thus the pseudocapsule becomes thick enough to become more noticeable.^33^ In tumors >4 cm, the tumors always increase and grow rapidly, leading to a thin pseudocapsule due to a short reactive hyperplasia period. Or it can also be explained that when the tumor size exceeds 4 cm, it always invades the renal capsule and infiltrates into perirenal fat so the pseudocapsule is disrupted or even absent.^34^

In our study, we also found that most malignant renal tumors show heterogeneous enhancement on CEUS in all three size groups. In contrast, most benign tumors in group I and II showed homogeneous enhancement, and in group III, two‐thirds of the benign tumors showed heterogeneity. This result can improve the value of tumor heterogeneity on CEUS for differentiating malignant and benign tumors ≤4 and >4 cm. Lee et al.^35^ reported that for tumors ≤3 cm, CEUS shows homogeneous enhancement regardless of the histological subtype. They hypothesized that the small tumors grow too slow to have necrosis change, while for a continually growing lesion with less blood supply, necrotic became frequently observed.^35^ This is not consistent with our finding among malignant tumors. This difference may be related to differences in the pathological grade of the involved tumors.

Our study had several limitations. First, this was a single institution study. And because ultrasound diagnosis is largely dependent on the clinical experience of physicians, inter‐observer inconsistency is inevitable; a large sample size multicenter study can further evaluate inter‐observer consistency. Second, since this study was a retrospective study of a large sample, quantitative analysis was not performed on some cases, so the value of quantitative analysis method of CEUS was not discussed in this study. Therefore, we will carry on a multi‐center study and explore the utility of quantitative analysis. It is expected this will make CEUS features more valuable to the differential diagnosis of benign and malignant RCC.

## 5 CONCLUSION

CEUS offers an option to characterize both tumor shape and enhancement pattern without ionizing radiation,^36^ and with, at least, a non‐inferior diagnostic performance compared with CECT and MRI. In this scenario, CEUS can reveal the perfusion characteristics of renal tumors of different size in real time, especially the presence of enhancement homogeneity and the pseudocapsule sign, thus providing valuable information for the differential diagnosis on CEUS between malignant and benign tumors.

## AUTHOR CONTRIBUTIONS

Yukun Luo, Qiuyang Li, and Jianing Zhu: Project development. Jianing Zhu, Luda Song: Data collection or management. Qiuyang Li, Nan Li and Yanjie Wang: Data analysis. Jianing Zhu: Manuscript writing. Ping Zhao, Luda Song and Qing Song: Statistical analysis.

## FUNDING INFORMATION

This study was supported by the National Natural Science Foundation of China (No. 81971635); the 2020 Industrial Technology Basic Public Service Platform Project of China (No. 2020‐0103‐3‐1); the Natural Science Foundation of Beijing (No. M22015).

## CONFLICT OF INTEREST

The authors report no conflicts of interest.

## Data Availability

N/A
